# Tumor Blood Flow Differs between Mouse Strains: Consequences for Vasoresponse to Photodynamic Therapy

**DOI:** 10.1371/journal.pone.0037322

**Published:** 2012-05-18

**Authors:** Rickson C. Mesquita, Sung Wan Han, Joann Miller, Steven S. Schenkel, Andrew Pole, Tatiana V. Esipova, Sergei A. Vinogradov, Mary E. Putt, Arjun G. Yodh, Theresa M. Busch

**Affiliations:** 1 Department of Physics & Astronomy, University of Pennsylvania, Philadelphia, Pennsylvania, United States of America; 2 Institute of Physics, University of Campinas, Campinas, Sao Paulo, Brazil; 3 Department of Biostatistics, University of Pennsylvania, Philadelphia, Pennsylvania, United States of America; 4 Department of Radiation Oncology, Perelman School of Medicine, University of Pennsylvania, Philadelphia, Pennsylvania, United States of America; 5 Department of Mathematical Sciences, New Jersey Institute of Technology, Newark, New Jersey, United States of America; 6 Department of Biochemistry and Biophysics, University of Pennsylvania, Philadelphia, Pennsylvania, United States of America; MGH, MMS, United States of America

## Abstract

Fluctuations in tumor blood flow are common and attributed to factors such as vasomotion or local vascular structure, yet, because vessel structure and physiology are host-derived, animal strain of tumor propagation may further determine blood flow characteristics. In the present report, baseline and stress-altered tumor hemodynamics as a function of murine strain were studied using radiation-induced fibrosacomas (RIF) grown in C3H or nude mice. Fluctuations in tumor blood flow during one hour of baseline monitoring or during vascular stress induced by photodynamic therapy (PDT) were measured by diffuse correlation spectroscopy. Baseline monitoring revealed fluctuating tumor blood flow highly correlated with heart rate and with similar median periods (i.e., ∼9 and 14 min in C3H and nudes, respectively). However, tumor blood flow in C3H animals was more sensitive to physiologic or stress-induced perturbations. Specifically, PDT-induced vascular insults produced greater decreases in blood flow in the tumors of C3H versus nude mice; similarly, during baseline monitoring, fluctuations in blood flow were more regular and more prevalent within the tumors of C3H mice versus nude mice; finally, the vasoconstrictor L-NNA reduced tumor blood flow in C3H mice but did not affect tumor blood flow in nudes. Underlying differences in vascular structure, such as smaller tumor blood vessels in C3H versus nude animals, may contribute to strain-dependent variation in vascular function. These data thus identify clear effects of mouse strain on tumor hemodynamics with consequences to PDT and potentially other vascular-mediated therapies.

## Introduction

Blood flow in tumors is a highly dynamic process that is commonly observed to fluctuate over time [1]. Fluctuations over timescales on the order of minutes or tens of minutes have been identified in the tumors of many animal models, as well as in human disease [1], [2], [3], [4]. These fluctuations, documented both at the individual and network blood vessel level [5], have been attributed to causes such as vascular intussusception from rapid vessel remodeling, locally-determined hemodynamics, and coordinated vasomotion under upstream control [3]. Importantly, temporal variations in tumor blood flow can affect therapy response, such as in the delivery of oxygen or drugs to the tumor [6]. Furthermore, tumor blood flow can reflect the extent of structural adaption by its vasculature [7], and therefore can provide potentially useful information about the developmental status of the tumor blood vessels and their capability for hemodynamic response.

Although infrequently considered in studies of tumor blood flow, the animal strain of tumor propagation is a possible variable of hemodynamic consequence. Some appreciation of the effect of animal strain on tumor blood flow and oxygenation can be gained from experiments that have examined these parameters as a function of tumor model. In a study by Guichard et al. [8], for example, SCCVII mouse tumors grown in C3H hosts experienced large decreases in blood flow after hydralazine administration (e.g. larger than analogous decreases in human tumors grown in nude mice), but the data revealed that the hydralazine effect in SCCVII tumors was smaller when implanted in nude versus C3H mice. In a related vein, Yasui et al. [9] reported that differences in pericyte density contributed to disparities in vascular function and oxygenation between tumor models grown in C3H versus nude murine hosts. Outside of the oncology field, a role for mouse strain in cardiovascular function and stress response is well documented [10], [11].

The studies presented herein were stimulated by an observation that vascular response during photodynamic therapy (PDT) of tumors differed between mouse strains. In PDT a photosensitizer and visible wavelengths of light are employed to cause local tissue damage, which for many protocols includes damage to blood vessels [12], [13]. This effect is evidenced by the substantial change and variability in tumor blood flow during the illumination period of PDT [13], [14], [15], [16], [17]. During PDT with the photosensitizer Photofrin, for example, tumor blood flow decreases during illumination at a rate that correlates with long-term tumor response [14]. Others have shown the luminal diameter of tumor blood vessels to progressively decrease during PDT, thus demonstrating that vasoconstriction contributes to decreases in blood flow during Photofrin-mediated treatment [18].

The effect of pre-existing tumor blood flow on vasoresponse to PDT has been considered by He et al. [19]. They showed that vessels with slower blood flow were more rapidly shut down after PDT. It has also been observed that tumor areas with low pre-existing pO_2_ experience larger decreases in tumor oxygenation after PDT [20]. This observation is consistent with the results of He et al., because it is likely that low pO_2_ areas suffered from low blood flow (at least temporarily). Indeed, correlations between oscillations in local tumor blood flow and fluctuations in pO_2_ have been reported [1], [5].

Our initial observation of strain-dependent decreases in tumor blood flow during PDT led us to the hypothesis that baseline tumor hemodynamic function differed between strains. Herein, we evaluate differences in the structure and function of tumor blood vessels between mouse strains through studies of the same tumor model, the radiation-induced fibrosarcoma (RIF), grown in its syngeneic host, C3H mice, or, alternatively, in nude mice. The results identify little-explored characteristics of baseline blood flow in tumors, including correlations between low frequency fluctuations (∼9–14 min periods) in tumor blood flow and animal heart rate. Moreover, when compared to tumor blood flow in nudes, tumor blood flow in C3H animals was more sensitive to physiologic or stress-induced perturbations. From these data we conclude that the underlying structure and hemodynamics of tumor blood vessels may inform upon the nature of their response to a subsequent vascular challenge.

## Materials and Methods

### Ethics Statement

All animal studies were approved by the University of Pennsylvania Institutional Animal Use and Care Committee and animal facilities are accredited by the American Association for the Accreditation of Laboratory Animal Care.

### Animals and tumor model

RIF tumors were propagated by the intradermal injection of 3×10^5^ cells over the shoulders of 9–11 week old female C3H (C3H/HeNCr) or nude (NCr-nu/nu) mice (NCI-Frederick, Frederick, MD). Animals were studied ∼7–10 days later when their tumors were ∼6–8 mm in diameter. The fur in C3H mice was clipped prior to cell inoculation and the treatment area depilated (Nair) at least 24 hours before monitoring with diffuse correlation spectroscopy and treatment with PDT.

### Experimental protocol

Throughout monitoring and PDT, mice were positioned on a heating pad set to 38°C (Gaymar Industries, Orchard Park, NY) and were anesthetized by inhalation of isoflurane in medical-air delivered through a nose-cone (VetEquip anesthesia machine, Pleasanton, CA). Concurrent with optical data acquisition, systemic physiological signals of heart rate (HR) and breath rate (BR) were recorded with a vitals monitor (MouseOx, Starr Life Sciences Corp., Allison Park, PA), through a sensor placed on the hind leg of the animal (acquisition rate of 1 Hz). In selected experiments, tumor and animal core temperatures were also measured via thermocouple (Omegaette HH306 DataLogger Thermometer and Thermoworks Microtherma thermometer with Braintree Type T Thermocouple Copper-Constantan rectal probe). Animals underwent one hour of baseline monitoring by diffuse correlation spectroscopy (see below) prior to PDT or L-NNA (N^G^-nitro-L-Arginine; Sigma, St. Louis, MO) injection, followed by continuous monitoring throughout PDT or 60 minutes after L-NNA injection. L-NNA was administered via the orbital plexus at a dose of 20 mg/kg (dissolved in saline). PDT was performed as described below. Photofrin concentrations in tumors were assayed as previously described [21].

### Photodynamic therapy

For photodynamic therapy (PDT) animals received an i.v. tail vein injection of Photofrin (available from Pinnacle Biologics, Bannockburn, IL) at 5 mg/kg and 24 h later microlens-tipped fibers (Pioneer, Bloomfield, CT ) were used to deliver 632±3 nm light from a Ceralas diode laser (Biolitec AG, Jena, Germany) over a 1.1 cm diameter field centered on the tumor. Laser output was measured with a LabMaster power meter (Coherent, Auburn, CA) and adjusted to deliver an irradiance of 75 mW/cm^2^ at the tumor surface. A total fluence of 135 J/cm^2^ was delivered to the tumor.

### Diffuse correlation spectroscopy

Tumor blood flow was continuously monitored with a noninvasive optical technique known as Diffuse Correlation Spectroscopy (DCS) [14], [22], [23]. Diffuse optical techniques employ photons in the near-infrared range that diffuse through tissue and can be detected millimeters to centimeters away from the source. DCS quantifies the temporal fluctuations of detected light. These fluctuations are sensitive to the motions of scatterers in tissue such as red blood cells. Therefore, the DCS signal provides a direct measure of blood flow, and can be used to derive a blood flow index (BFI) parameter. This technique is used herein to derive microvascular relative blood flow (rBF), i.e. blood flow changes relative to a baseline period (rBF(t) = BFI(t)/BFI_baseline_). The baseline was taken as mean BFI over the one hour of monitoring at each source-detector combination. Tumor rBF variations due to PDT were calculated by normalizing to the BFI obtained two minutes before beginning of illumination. Similarly, tumor rBF changes due to L-NNA were calculated by normalizing to the BFI obtained during the last two minutes of the baseline period.

DCS measurements were performed with a home-built instrument containing one continuous-wave, long coherence length, 785 nm laser (CrystaLaser Inc., Reno, NV), and four single-photon counting avalanche photodiodes (PerkinElmer, Canada). The laser source was coupled to an optical switch with nine outputs to provide information from up to 36 source-detector combinations designed to cover the whole tumor area. Both source and detector optical fibers were arranged in a two-dimensional pattern inside a non-contact probe composed of a camera and neutral density optical polarizing filter. The lens of the camera was positioned about 15 cm above the tumor. The separations between a source and a detector position on the surface of the tumor ranged from 0.97 to 4.37 mm. The detectors fed a 4-channel autocorrelator board (Correlator.com, Bridgewater, NJ) that computed the temporal intensity autocorrelation function. The whole cycle took approximately 16 s.

Tumor rBF for each source-detector combination was obtained by fitting the temporal intensity autocorrelation functions to a semi-infinite homogeneous medium diffusion model that approximates the mean-square displacement of the scatterers in tissue as a Brownian motion in a semi-infinite homogeneous medium [23], [24]. The mean tumor rBF of each animal was calculated by averaging tumor rBF over all the source-detector combinations, and is displayed as a percentage of values obtained during the baseline period.

### Phosphorescence lifetime measurements

Phosphorescence lifetime measurements were employed to measure tumor oxygenation during the one-hour baseline period. The measurement generally followed our recently published protocol [25]. Oxyphor G4 (20 µl of 20 µM solution) was injected directly into the tumor interstitial space and phosphorescence was measured using a fiber-optic phosphorometer [26] modified for time-domain operation. Phosphorescence was excited by 10 **µ**s-long LED pulses (**λ**_max_ = 635 nm) and decays were collected over 3 ms period. Typically, 200 decays were averaged for a single measurement, and the measurements were recorded every 4 s. The data were analyzed by way of single-exponential fitting and subsequent conversion of the phosphorescence lifetime to pO_2_ using appropriate calibrations [25].

### Immunohistochemistry

In tumors from C3H and nude animals immunohistochemistry was used to quantify blood vessels and smooth muscle actin (SMA) expression. Frozen tumors preserved in Tissue Tek OCT Compound (Sakura Finetek, Torrance, CA) were sectioned (14 **µ**m) on a Microm HM 505E Cryostat (Microm International GmbH, Walldorf, Germany) and fixed in graded solutions of ethanol (70%, 85% and 100% at 4°C, followed by 100% at room temperature). Following a previously published procedure for CD31 staining [27], vascular structure was labeled with a CD31 antibody (1∶100 for 1 h; BD Pharmingen, San Diego, CA) and vessel maturity with an antibody to smooth muscle actin (1∶100 for 1 h; GeneTex, Irvine, CA). Respective to these primary antibodies, secondary antibodies of Cy5-conjugated mouse anti-rat (1∶50 for 45 min; Jackson Immunoresearch, West Grove, PA) and FITC-conjugated donkey anti-rabbit (1∶200 for 1 h; Jackson Immunoresearch) were used. Sections were flooded with Hoechst 33342 (20 µM) to label tissue, and images (10×) were collected by fluorescence microscopy using a Nikon Eclipse 800 microscope (Melville, NY) with a motorized stage (Ludl electronics; Hawthorne, NY) and 100-W high pressure mercury arc lamp and Photometrics Quantix CCD digital camera (Tucson, AZ). Analysis entailed masking of CD31, SMA and Hoechst-stained areas; identification of CD31 “objects” based on contiguous staining; and calculation of “object” size, as well as the percentage of tumor positive for CD31 or SMA. All steps of this analysis were performed using routines in the Image toolbox in MATLAB (MathWorks, Natick, MA) with the exception of masking for SMA and Hoechst, which utilized Adobe Photoshop (Adobe Systems Inc., San Jose, CA). Controls included slides stained with secondary (but no primary) antibody, and demonstrated no staining.

### Data analysis and statistics

Hemodynamic time-series during PDT were averaged over all the source-detector pairs. rBF response due to PDT was estimated by the difference between two reference points in the temporal rBF time-series: the point when rBF initially declined and the point when it plateaued. These “change points” were found based on the likelihood ratio method. The L-NNA induced decrease was reported as the lowest rBF level between 10 and 40 minutes after L-NNA administration.

For the one-hour monitoring prior to any intervention, trends were obtained by fitting the raw time-series by a cubic polynomial to capture the global trend. Fluctuations in tumor rBF were quantitatively analyzed by calculating the autocorrelation function (ACF) of the de-trended time-series (i.e. the residuals after removing the global trend). For each mouse, quantification of the cyclic patterns was performed by averaging the first 3 time lags of the ACF (equivalent to approximately a 1-minute window), then taking the median of this number over all the source-detector pairs.

In order to investigate the relationship between tumor rBF and mouse physiology (assessed by HR or BR), we calculated the cross-correlation between the two raw time-series, discarding the first 5 minutes of the steady-state period to avoid initial instability. The acquisition time for each of 36 tumor rBF series was matched with the physiological data, which were then down-sampled to match the tumor rBF acquisition rate by averaging HR/BR over the appropriate time interval. The maximum cross-correlation value across the time series was used to quantify the degree of correlation between mouse physiology and tumor rBF.

In all the procedures above, tumor rBF for each source-detector combination was analyzed independently. For each animal, the median tumor rBF response was calculated by taking the median over all source-detector combinations. The data for each group of animals were summarized using either the median and the interquartile range (IQR), or the mean and the standard deviation (SD). Wilcoxon signed rank tests were used to assess statistically significant differences between the tumors of different strains, as well as within the tumors of each strain at different time points.

## Results

### PDT-induced reductions in tumor blood flow differ in magnitude between C3H and nude mice

**Figure 1:**
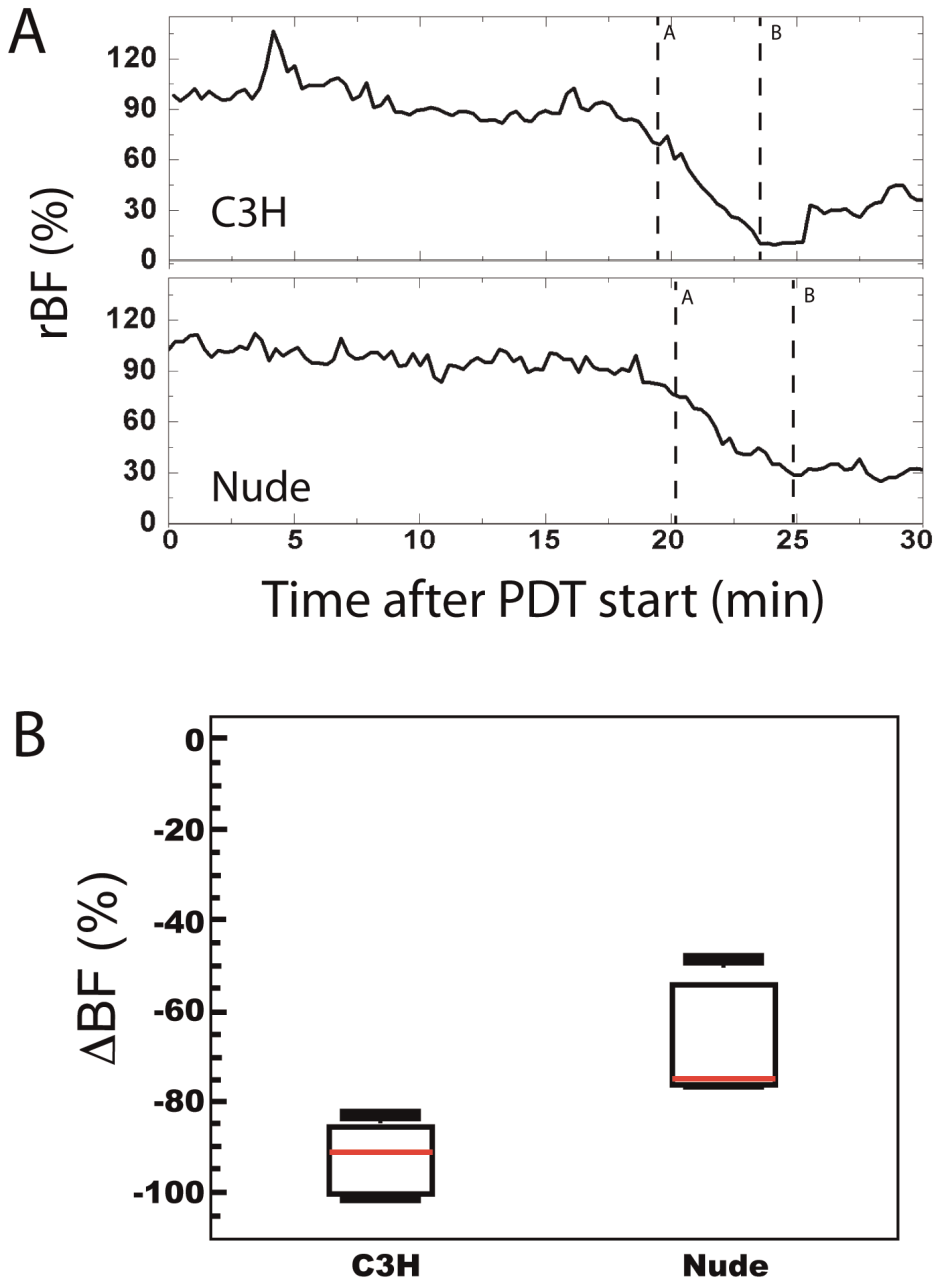
Strain-dependent differences in tumor blood flow during PDT. (A) Representative rBF time-courses in RIF tumors for a C3H and a nude mouse during 30 minutes of illumination with PDT (t = 0). The lines A and B represent the change points marking the beginning of the decrease in rBF and the plateau in rBF level, respectively, and were used to define the change in rBF (i.e. **Δ**BF = rBF_A_−rBF_B_). (B) Box plots of blood flow changes (**Δ**BF) during PDT within animals of each strain (N = 7 for each strain); PDT decreased rBF in both groups (p = 0.016 for both C3H and nudes), with the decrease in C3H larger than that in nudes (p = 0.026).

We have previously demonstrated that tumor blood flow markedly decreases during the illumination period for Photofrin PDT [14]. We confirm these results in the present investigation; a representative trace of rBF in RIF tumors of C3H mice is plotted in Fig. 1A (upper panel). RIF tumors of nude hosts (lower panel) also experience decreases in blood flow during PDT. Notably, however, the decrease in tumor rBF in nude animals is significantly less than that in C3H mice bearing the same tumor line; the median (IQR) PDT-induced decrease in blood flow is −91% (−97,−86%) in C3H compared to −75% (−76,−54%) in nudes (p = 0.026; Fig. 1B). No differences in Photofrin uptake were detectable between RIF tumors excised from the two mouse strains, and thus drug uptake does not account for strain-dependent vascular response (mean ± SD of Photofrin uptake is 3.63±1.92 and 3.83±1.60 ng/mg in C3H and nudes, respectively). These observations led us to hypothesize that underlying vascular function differed across the tumors of C3H and nude animals. To test this hypothesis, we studied tumor blood flow in anesthetized, but otherwise unperturbed animals of each strain. For analysis, the blood flow patterns were separated into two components: long term trends and cyclic patterns (i.e. fluctuations around the trends).

### Murine tumors demonstrate cycles in blood flow that depend on mouse strain

**Figure 2:**
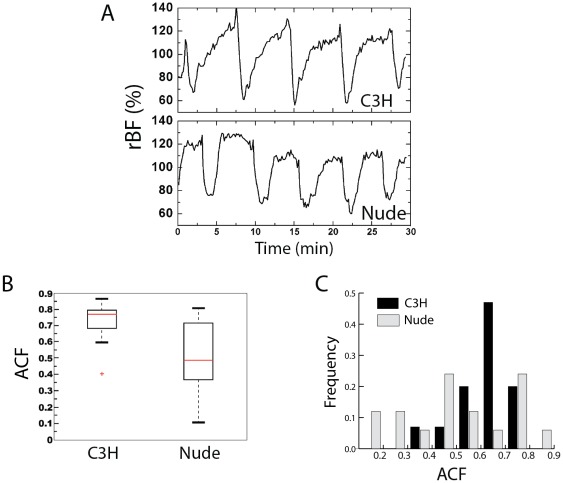
Cyclic tumor blood flow at baseline depends on mouse strain. (A) Representative rBF time-courses in RIF tumors for a C3H and a nude mouse during one hour of unperturbed monitoring. (B) Mean autocorrelation function (ACF) across signal-detector pairs of the first three lags (equivalent to 1 minute) for each strain; p = 0.003 for the difference between the strains. (C) Histogram of the distribution of the mean ACF among the mice of each strain. (N = 17/15 for nudes/C3H).

Blood flow in tumors can fluctuate in repetitive cycles of various frequencies attributable to the effects of vasomotion or local vascular structure [3]. Over the course of one hour of monitoring, such cycles were clearly visible in selected animals of both the C3H and nude strains (Fig. 2A). When considering all of the animals of a given strain, it became apparent that the consistency of cycling differed between the strains. C3H mice were characterized by more well-defined and regularly-cycling tumor blood flow than nudes. The regularity of the blood flow oscillations is readily quantified using the autocorrelation function (ACF), which essentially provides a measure of the correlation between two sequentially observed blood flow values (traces) from the same animal as a function of the time lapse (or time lag) between the observations. If the cyclic pattern in rBF is clear, and if the oscillation period is well defined, then the ACF will decrease smoothly as the time lag increases. On the other hand, a lack of periodicity leads to a quick drop of the ACF after the very first time lag. Across animals, the median (IQR) ACF value was 0.77 (0.69,0.79) in tumors of C3H mice over 60 minutes of monitoring; this median ACF is significantly higher (p = 0.003) than the median ACF of 0.49 (0.39,0.71) for tumors in nude animals (Fig. 2B). The higher ACF in the C3H group reflects a more pronounced periodic behavior of tumor rBF when compared to the nude group.

In addition to demonstrating more regularity in blood flow *within their tumors*, in comparisons *among animals*, C3H mice were more likely to host tumors with cyclic blood flow than their nude counterparts; thus, cycling tumor blood flow was more prevalent among C3H than nude animals. This observation is quantifiable through the distribution of the ACF among the animals of each strain. While most of the C3H animals presented with ACF>0.6, the ACF in the nude mice was distributed throughout a range from 0.1 to 0.9 (Fig. 2C), wherein the lowest values correspond to animals that essentially showed no periodicity. Overall, 2 of the 17 animals in the nude group (12%) exhibited no visibly clear pattern in their temporal patterns of flow. Among animals in the nude group that did demonstrate visible regular oscillations, the median (IQR) period length was 9.1 (7.8, 10.9) min. In the tumors of C3H mice, all animals demonstrated a regular oscillation with a median length of 13.6 (10.9,18.1) min. Thus among animals that demonstrated periodic blood flow, that period length was similar between the strains; however, strain-dependent differences were evident by the fact that all the C3H animals exhibited regular periodicity while 12% of nudes did not.

### Cycles in tumor blood flow associate with animal heart rate in both strains

**Figure 3:**
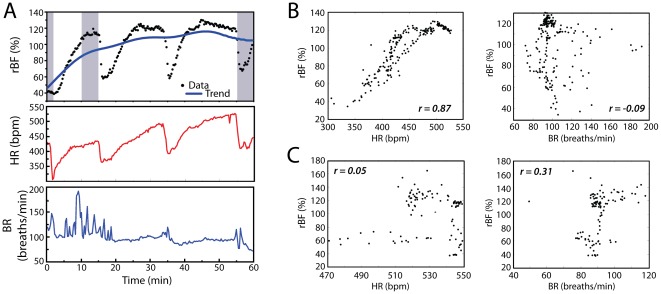
Tumor blood flow is closely associated with animal heart rate. (A) Representative time-courses of tumor rBF, heart rate (HR) and breath rate (BR) for a C3H animal during one hour of unperturbed monitoring. The shadowed areas represent the time periods for trend analysis presented in Table 1, while the blue line shows the fitted global trend line. (B) For the animal in “A”, scatter plots (at time lag 0) depict the association between rBF and HR (left) or rBF and BR (right) during the one hour of baseline monitoring or (C) during PDT. The *r* values in the scatter plots represent the maximum cross-correlation value between the two variables.

Since the present investigation identified synchronized, tumor-wide blood flow changes (i.e. the volume over which DCS averaged), we sought a systemic cause for the oscillations. Fig. 3A shows representative time-courses of rBF, HR and BR for a single anesthetized C3H mouse over a 1-hour period. The similarity of the temporal fluctuations in tumor blood flow and the mouse heart rate is evident. On the other hand, fluctuations in tumor rBF do not appear to be temporally related to fluctuations in the mouse breath rate. For this animal, scatter plots depict the close relationship between HR and tumor rBF, and the absence of such a relationship between BR and tumor rBF (Fig. 3B). Indeed, the cross-correlation coefficient between rBF and HR (BR) yielded 0.87 (−0.09) for this specific mouse. Across animals, the median (IQR) correlation coefficient between rBF and HR was 0.51 (0.35,0.67) for C3H and 0.49 (0.39,0.74) for nudes. Both values are higher than zero (p<0.001), indicating a significant association between tumor rBF and mouse HR for both strains. In contrast, tumor rBF and mouse BR showed a very weak correlation with cross-correlation coefficients of 0.25 (0.14,0.32) and 0.29 (0.13,0.39) in C3H and nudes, respectively.

It is worth noting that the association between tumor blood flow and animal heart rate was decoupled by the local vascular stress of PDT. In both strains, PDT-induced change in tumor blood flow served to override its dependence on animal HR. Representative examples show both heart rate and breath rate to be poorly correlated with tumor rBF during PDT (Fig. 3C); across animals, the median cross-correlation between tumor rBF and mouse HR was not significantly different from zero in either model, being 0.22 (−0.40,0.57) and 0.10 (−0.07,0.16) in C3H and nudes, respectively.

### Long-term trends in tumor blood flow demonstrate increases in C3H mice

To assess overall trends in tumor blood flow over time, rBF in anesthetized animals was compared between three defined periods during 1 hour of monitoring: initial rBF (average of the first 0–2 minutes), short-term rBF (average of 10–15 minutes into monitoring), and equilibrated rBF (average of 55–60 minutes into monitoring). These timeframes of comparison are shown on Fig. 3A, along with a representative example of a global trend line fit to the data. Table 1 summarizes the median changes in rBF trends for these time ranges in both strains. Overall, the C3H group showed a median (IQR) significant increase of 26% (18,39)% in rBF over the 60-minute period (p = 0.0001). However, this increase was not constant over time, since half of it happened within the first 15 minutes of monitoring, while the remaining change occurred over the next 45 min. In contrast, tumor rBF did not change significantly in the nude group, with a slight median increase of 9% (−11,29)% over 60 minutes.

**Table 1:** Strain-dependent changes in rBF trends at three different time periods during one hour of unperturbed monitoring.

Strain	Time Ranges (min)	Median (IQR) changes (%)	Significant change from zero (p-value)
C3H	15 vs. 2	13 (10, 25)	0.0001
	60 vs. 2	26 (18,39)	0.0001
	60 vs. 15	11 (4,19)	0.003
Nude	15 vs. 2	1 (−6,9)	0.49
	60 vs. 2	9 (−11,29)	0.28
	60 vs. 15	6 (−8,12)	0.21

Baseline level of rBF increases in C3H animals, but is stable in nudes.

Accompanying these strain-dependent differences in tumor blood flow over the 60 min period were strain-dependent differences in mouse heart rate. However, neither tumor nor animal core temperature changed in either strain. Over 60 minutes, C3H heart rate increased significantly (p = 0.0001) from a median (IQR) of 426 (405,436) beats per minute to 491 (459,515) beats per minute. In contrast, the change in nude heart rate from 428 (394,454) beats per minute to 442 (350,464) beats per minute was not significant (p = 0.82). Mean (SD) tumor temperature remained steady in both strains, measured at 27.3 (1.1) °C during the first two minutes and 28.3 (1.3) °C during the last five minutes in C3H and at 27.9 (1.2) °C during the first two minutes and 28.8 (0.7) °C during the last five minutes in nudes. Similar non-significant changes were found in core temperature, with values changing from 31.2 (1.5) °C to 32.1 (1.1) °C in C3H and from 32.2 (1.5) °C to 32.8 (1.1) °C in nudes. Thus, there was no evidence of temperature contributing to the differential blood flow responses between nudes and C3H, but strain-dependent change in heart rate is aligned with the blood flow change.

**Figure 4:**
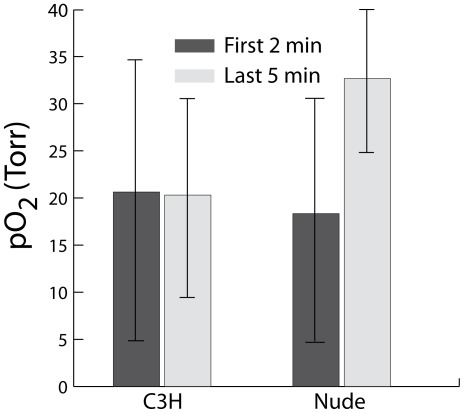
Tumor oxygenation is not significantly different between the two strains. Tumor oxygenation (mean ± SD) in animals of each strain for measurements made at the beginning and the end of a one-hour period. Oxygen concentrations measured by phosphorescence lifetime monitoring. (N = 6 for each strain.)

Given the increases in C3H tumor blood flow, we speculated that tumors of C3H animals may be more oxygenated than those of nude animals. Because PDT is oxygen-dependent, this could facilitate the stronger PDT-mediated vascular response found in C3H (see Fig. 1). To study this possibility, phosphorescence lifetime measurements were used to assess tumor oxygenation in C3H and nude animals (Fig. 4). Average (SD) tumor oxygen tension in the first two minutes of anesthesia was 18 (13) Torr and 21 (16) Torr in nudes and C3H, respectively, which changed to 33 (10) Torr in nudes and 20 (12) Torr in C3H at the conclusion of 60 min. There is substantial overlap among these values, both between the animal strains at a given time and between the time points in a given strain. These data show that RIF tumors in C3H mice were not better oxygenated than those in nude mice on an absolute basis over the 1-hour period (p = 0.13), nor did their tumor oxygen tension increase in a statistically significant fashion over the monitoring period (p = 1 and 0.07 for C3H and nude animals, respectively). Trends of increasing blood flow in the C3H animals did not lead to differentially better oxygenation of the tumors in these animals compared to those of the nudes.

### Response to vasoconstrictor L-NNA also differs between strains

**Figure 5:**
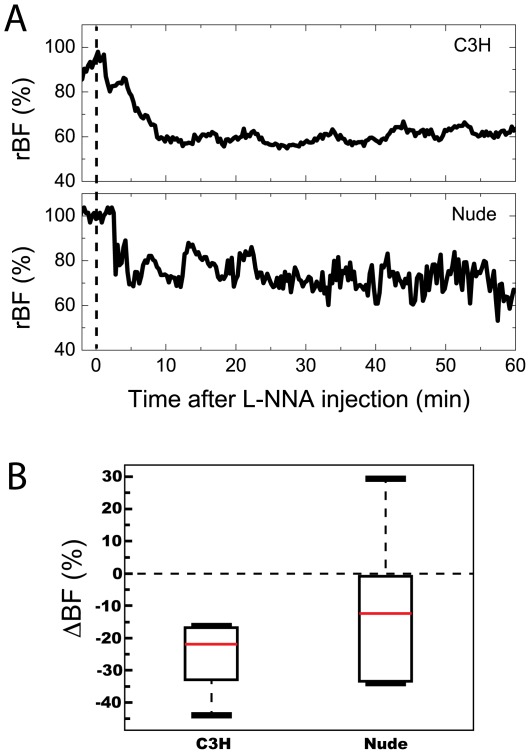
L-NNA decreases tumor blood flow in C3H animals. (A) Representative rBF time-courses in RIF tumors for a C3H and a nude mouse after L-NNA administration (represented by the dashed line at *t = 0*). (B) Box plots of blood flow changes (**Δ**BF) after L-NNA administration within animals of each strain (N = 7 each); L-NNA decreased tumor rBF within C3H (p = 0.016), but not within nude animals (p = 0.16). As with previous experiments, nude animals displayed substantial heterogeneity in **Δ**BF.

In order to explore the generality of strain effects on vascular dynamics, we next evaluated rBF response to vasoconstriction induced by nitric oxide synthase inhibition with L-NNA. The typical time-course of LNNA-induced blood flow reduction is shown in representative traces from tumors in a C3H and a nude mouse (Fig. 5A), and from these the maximum L-NNA induced decrease was measured in a 30 min window between 10 to 40 min after drug injection. This analysis revealed tumor rBF to significantly decrease after L-NNA administration in the C3H group, with a median (IQR) decrease of −22% (−33,−17%) (p = 0.016). On the other hand, nude animals experienced a wider range of responses to L-NNA, leading to a non-significant (p = 0.16) change of −12% (−33,−1%) in tumor rBF(Fig. 5B).

### Strain-dependent differences in tumor vascular structure

**Figure 6:**
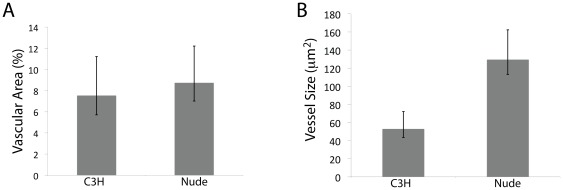
Tumor vascular structure is also strain-dependent. (A) Vascular area (as a percent of the tumor) and (B) median vessel size in the tumors of animals of each strain. Vessels were stained by immunochemistry for CD31 in sections collected from each of 4 and 5 tumors from C3H and nude mice, respectively; p = 0.02 for the difference in vessel size between the models. Error bars indicate standard deviations.

Taken collectively, the above data find the hemodynamics of RIF tumors in C3H mice to be more tightly regulated, as shown by its regular cyclic patterning and reactivity to both local (PDT) and systemic (L-NNA) vascular stress. These results could be reflective of an underlying difference in the vascular makeup of the tumors, leading us to compare the vascular composition of RIF tumors grown in C3H versus nude animals. Vascular areas were similar between the tumor models. On average (SD), RIF tumors in C3H mice had a vascular area of 7.5 (3.7)%, compared to a vascular area of 8.7 (3.5)% in the nude animals. In contrast, though, the size of the tumor blood vessels differed significantly between strains. In C3H mice median vessel size was an average (SD) of 53 (19) µm^2^. In nudes the tumor blood vessels were significantly larger (p = 0.02) at 130 (33) µm^2^ (Fig. 6).

## Discussion

Vascular damage is an important mechanism of PDT action with many photosensitizers and photosensitizing conditions. The extent of this damage can be expected to vary as a function of photosensitizer type, photosensitizer dosing, drug-light interval, fluence, and fluence rate [28], [29], [30], [31]. However, the effect of animal strain on PDT-induced vasoresponse has barely been considered. This led us to investigate how tumor hemodynamics during PDT differed between RIF tumors as a function of their propagation in C3H versus nude mice. Tumors of C3H mice developed more severe ischemia during PDT, and this finding could not be attributed to differences in either photosensitizer uptake or pre-existing oxygen tensions between tumors of the two strains. In contrast, tumor vessels of C3H mice were smaller than those in nudes and demonstrated more tightly controlled hemodynamics, evidenced by the presence and regularity of cyclic blood flow patterns; cyclic patterns were absent or not as well defined in the larger tumor blood vessels of the nude mice. Our data show that these strain-dependent differences in tumor hemodynamics have implications in studies of PDT. These differences may also play a role in response to systemic but less severe vascular insults, such as the vasoconstrictor L-NNA, which led to significant decreases in tumor blood flow in C3H but not nude mice.

A role for mouse strain in the response of tumor blood flow to vasoactive drugs has previously been unclear. A handful of papers have considered this possibility with the conclusion that animal strain did not play a role because differentials in blood flow response between strains were small compared to differences between tumor models [8], [32]. Nevertheless, the data showed drug-induced changes in blood flow to be slightly bigger for the same tumor model grown in C3H mice versus nude or SCID animals. Laser Doppler was used to measure tumor blood flow in these studies resulting in a sampled area that was small (∼1 mm^3^) compared to the tumor-wide average provided by DCS in the present study. Given the known intra-tumor heterogeneity in tumor vascularization and its reactivity these differences in sampling size could readily explain the more clear-cut results of the present study.

The immune system is a factor that should be considered when comparing vascular damage between nude and C3H animals. Propagation of the same tumor model in two different murine strains necessitated that one of theses strains be immunodeficient. Previous reports have shown that the efficiency of PDT is compromised in immunodeficient hosts [33]. The fact that C3H mice have an intact immune system, while nude mice are athymic and immune deficient, may account for some of the strain-dependent differences due to PDT. However, it is unlikely that the differences we found were a result of differences in T-cell response to PDT because our studies were limited to changes in blood flow during the course of PDT (30 minute treatment), which is likely earlier than the consequences of T-cell deficiency on PDT outcomes would be realized in the nude animals.

On the other hand, immunological-dependent differences in strain responses could be mediated through its effect on tumor development. Solesvik et al. [34] have documented differences in the vascular composition of multiple tumors lines when propagated in nude versus C3H hosts. Specifically, in the tumors of C3H mice, small diameter (∼10 µm) blood vessels were significantly longer, in fact sometimes twice as long, as blood vessels of similar diameter in the tumors of nudes. Given the tortuous nature of tumor vascular networks, a vessel can transverse a 2D plane multiple times and the longer that a vessel is the more likely it is that this will happen. Thus, the increased length of small diameter blood vessels in the tumors of C3H animals could contribute to our finding that C3H mice bore tumors with smaller-sized vessels. Solesvik et al. did not consistently detect decreases in vessel diameter in C3H versus nude mice across all tumor models studied, but some did indeed show trends toward decreased diameter in the C3H animals (and the RIF model was not among those investigated).

Potential factors contributing to differentials in vessel size between nudes and C3H may be found in studies of the effects of immunogenicity on tumor-associated fibrosis and interstitial fluid pressure. Nude animals lack the lymphocyte-laden fibrosis associated with tumors in immune competent mice [35]. Given the stronger fibrotic reaction in immune competent animals, which could increase cell density surrounding the blood vessels, it comes as no surprise that others report tumor interstitial fluid pressure to be higher in immune competent mice than in the same tumor model when grown in nude animals [36]. Our findings of smaller-sized vessels in the C3H versus nude animals would therefore be in agreement with expected differentials in tumor interstitial fluid pressure as a function of immunogenicity. Namely, vessel compression could result from high interstitial fluid pressure in immune competent animals, while lower interstitial fluid pressures in the tumors of immune deficient animals could be permissive of larger-sized vessels. Taken together these data point to vascular structure as a variable to be considered in studies of tumor response to vasomodulation within different murine strains.

Differences in vessel size between the tumors of C3H and nudes could contribute to the strain-dependent differences in vasoresponse due to the effect of vessel size on blood flow. Resistance to blood flow is inversely related to the fourth power of vessel diameter [7], predicting that vasoconstriction of a small vessel would increase flow resistance to a greater magnitude than the same vasoconstrictive insult in larger vessels (Poiseuille's law assuming vessel radius decreases by the same amount in each case). Under the assumptions that the concentration of blood vessels is the same in the two models (data show that the vascular area is the same) and that blood pressure is the same (or changes in the same way) between the models, one can then calculate that vasoconstriction of the smaller tumor blood vessels in C3H mice would lead to larger relative changes in blood flow than would the same amount of vasoconstriction in the larger vessels of nude mice. Indeed, PDT-created decreases in tumor rBF were significantly larger in C3H animals. Moreover, the vasoconstrictor L-NNA significantly decreased tumor blood flow in C3H animals, while resulting in smaller, more variable and overall insignificant decreases in tumor rBF in the nudes.

Our findings of oscillations in tumor blood flow under baseline conditions are consistent with many previous reports of fluctuating blood flow or oxygen levels in tumors [2], [4], [6], [37], [38], [39]. Such fluctuations have even been noted in studies of PDT along with the observation, as we made, that they are destroyed by the treatment [20]. Factors such as tumor size or vessel maturity (expression of smooth muscle actin, SMA) [9], [40] can affect the nature of the fluctuations, but these characteristics did not differ between the tumors of C3H versus nudes in our study: mean (SD) tumor size was 270 (88) mm^3^ vs. 292 (61) mm^3^ and SMA expression was 0.77 (0.36)% vs. 0.56 (0.58)% in C3H vs. nudes, respectively. The underlying cause of cyclic blood flow or hypoxia has been attributed to several factors, including vasomotion linked to upstream circulation, the local hemodynamics of blood flow through tortuous tumor vasculature and vascular intussusception from rapid vessel remodeling [3], [41], [42]. Our studies do not rule out contributions from the latter two factors, but the fact that flow cycled over the tumor as a whole (i.e. the area over which DCS measured) suggests a less-localized cause for the fluctuations. Vasomotion could provide one explanation. Using window chamber models, others have documented vasomotion to occur when the diameter of tumor-feeding arterioles produced coordinated changes in vessel diameter and blood flow through downstream daughter vessels [43]. Looking more generally, we have shown in the present study that the low frequency fluctuations in tumor blood flow are highly correlated with animal heart rate.

The median period length of cyclic tumor blood flow in this study was ∼9 and 14 min in nude and C3H mice, respectively, which is in good agreement with period lengths detected in fluctuating blood flow or oxygenation in tumors of other investigations [3], [43], including those in humans [44]. Given our findings that these fluctuations correlate with changes in animal heart rate, it is interesting to note that this period is similar between tumors and/or strains as reported herein by us and in the observations of others [45] because it supports the contribution of a more general-acting effector, such as heart rate, to fluctuation in tumor oxygenation or blood flow. Importantly, spontaneous fluctuations in murine heart rate with a similar period have been observed [46].

Strain-dependent differences in baseline hemodynamic function were evident in both cyclic fluctuations and longer-term trends. At the level of blood flow cycling, tumors of C3H had much more regular periods, evidenced by significantly higher autocorrelation functions than those found in the nudes. The C3H also were more likely to exhibit periodic fluctuations in tumor rBF and these fluctuations were completely absent from some of the nudes. In terms of longer term (over 1 hour) trends in tumor rBF, the C3H, but not the nudes, exhibited increasing blood flow over an hour of monitoring. Accompanying increases in heart rate were also detected in these animals. We suggest that these increases are related to the known vasodilative effect of isoflurane. Whereas we showed tumor rBF (C3H mice) to increase by ∼25% in this study, Schumacher et al. reported isoflurane to induce a 26% increase in the diameter of microvessels of rat skeletal muscle [47]. Neither tumor nor core animal temperature increased over this same monitoring period; moreover they did not differ between the strains, which shows that in the context of our investigations strain-dependent differences in tumor temperature did not exist or contribute to the differential in long term blood flow trends between the models.

Due to the anesthesia-induced increase in blood flow in the C3H animals, phosphorescence lifetime imaging was used to compare the oxygenation of tumors in C3H versus nude animals. Average (SD) tumor pO_2_ after 60 minutes of monitoring, i.e. the time point at which PDT would begin, was 20 (12) Torr in C3H and 33 (10) Torr in the nudes, showing substantial overlap between the strains. Thus, the stronger vascular effects of PDT in C3H mice could not be attributed to an improved state of oxygenation relative to the tumors in nude mice. A difference in Photofrin uptake between RIF tumors of nude and C3H animals was also ruled out as a cause of strain-dependent differences in PDT response.

In summary, comparative studies of the same tumor line grown in C3H and nude mice has found blood flow in the C3H animals to be more responsive to vascular stress, whether it be locally (PDT) or systemically (L-NNA) induced. Specifically, PDT uncouples cyclic fluctuations in tumor blood flow from animal heart rate, leading to decreases in tumor blood flow that are significantly greater in the C3H versus nude animals. These results may in part be explained by the smaller blood vessels of tumors in C3H mice, which could contribute to the general tighter control of blood flow in these tumors. All told, these results provide evidence that the underlying structure and hemodynamics of tumor blood vessels may inform upon the nature of their response to a subsequent vascular challenge. Differences in baseline tumor hemodynamics between animal strains should be considered when planning and interpreting studies of PDT, or other vaso-modulating applications.
